# Sex Differences in the Ventral Tegmental Area and Nucleus Accumbens Proteome at Baseline and Following Nicotine Exposure

**DOI:** 10.3389/fnmol.2021.657064

**Published:** 2021-07-14

**Authors:** Angela M. Lee, Mohammad Shahid Mansuri, Rashaun S. Wilson, TuKiet T. Lam, Angus C. Nairn, Marina R. Picciotto

**Affiliations:** ^1^Department of Psychiatry, Yale University, New Haven, CT, United States; ^2^Yale Interdepartmental Neuroscience Program, New Haven, CT, United States; ^3^Yale/NIDA Neuroproteomics Center, New Haven, CT, United States; ^4^Molecular Biophysics and Biochemistry, Yale University School of Medicine, New Haven, CT, United States; ^5^W.M Keck Biotechnology Resource Laboratory, Yale University School of Medicine, New Haven, CT, United States

**Keywords:** nicotine, proteomics, sex differences, ventral tegmental area, nucleus accumbens

## Abstract

Sex differences in behaviors relevant to nicotine addiction have been observed in rodent models and human subjects. Behavioral, imaging, and epidemiological studies also suggest underlying sex differences in mesolimbic dopamine signaling pathways. In this study we evaluated the proteome in the ventral tegmental area (VTA) and nucleus accumbens (NAc) shell in male and female mice. Experimental groups included two mouse strains (C3H/HeJ and C57BL/6J) at baseline, a sub-chronic, rewarding regimen of nicotine in C3H/HeJ mice, and chronic nicotine administration and withdrawal in C57BL/6J mice. Isobaric labeling with a TMT 10-plex system, sample fractionation, and tandem mass spectrometry were used to quantify changes in protein abundance. In C3H/HeJ mice, similar numbers of proteins were differentially regulated between sexes at baseline compared with within each sex after sub-chronic nicotine administration. In C57BL/6J mice, there were significantly greater numbers of proteins differentially regulated between sexes at baseline compared with within each sex after chronic nicotine administration and withdrawal. Despite differences by sex, strain, and nicotine exposure parameters, glial fibrillary acidic protein (GFAP) and dopamine and cAMP-regulated phosphoprotein of 32 kDa (DARPP-32, Ppp1r1b) were repeatedly identified as significantly altered proteins, especially in the VTA. Further, network analyses showed sex- and nicotine-dependent regulation of a number of signaling pathways, including dopaminergic signaling. Sub-chronic nicotine exposure in female mice increased proteins related to dopaminergic signaling in the NAc shell but decreased them in the VTA, whereas the opposite pattern was observed in male mice. In contrast, dopaminergic signaling pathways were similarly upregulated in both male and female VTA after chronic nicotine and withdrawal. Overall, this study identifies significant sex differences in the proteome of the mesolimbic system, at baseline and after nicotine reward or withdrawal, which may help explain differential trajectories and susceptibility to nicotine addiction in males and females.

## Introduction

Smoking tobacco is responsible for more than 7 million deaths a year worldwide^1^ and nicotine is the primary psychoactive ingredient in tobacco (Picciotto and Kenny, 2013). Like other drugs of abuse, one of the primary mechanisms contributing to the initial stages of nicotine reward is the ability of nicotine to increase phasic dopamine release from ventral tegmental area (VTA) projection neurons to the nucleus accumbens (NAc; Schultz, 2010; Nestler, 2005; Di Chiara and Imperato, 1988). Nicotine exerts its actions via activation and desensitization of nicotinic acetylcholine receptors (nAChRs), which are pentameric cation channels made of α (α2–α10) and β (β2–β4) subunits. nAChRs are expressed widely, with significant expression in multiple cell types in the VTA (Picciotto et al., 2008; Dani, 2015; Picciotto and Kenny, 2013).

Importantly, there are significant sex differences in many aspects of nicotine addiction, ranging from the cellular to behavioral levels. In clinical studies, women are not only more susceptible to developing nicotine addiction (Pogun et al., 2017; Pogun and Yararbas, 2009; Sylvestre et al., 2018), but also experience more difficulty quitting and higher rates of relapse (Weinberger et al., 2014; Smith et al., 2015, 2016). In preclinical studies, female rats self-administer more nicotine than male rats (Flores et al., 2019) and acquire self-administration more quickly than males at lower doses of nicotine (Donny et al., 2000). Further, nicotine interacts differentially with sex hormones in males and females to affect nicotine-induced dopamine release in striatal tissue in opposite directions (Dluzen and Anderson, 1997). Even the upregulation of α4β2 nAChRs, a classic response to chronic nicotine exposure, is less pronounced in female rodents (Koylu et al., 1997; Mochizuki et al., 1998; Hoegberg et al., 2015) and humans (Cosgrove et al., 2012) compared to their male counterparts. Significant sex differences in baseline function of the mesolimbic dopamine system have also been demonstrated, including the numbers of putative dopaminergic neurons in the VTA (McArthur et al., 2007), baseline VTA dopaminergic activity (Calipari et al., 2017), and D1 and D2-type dopamine receptor availability in the NAc shell and VTA (Pohjalainen et al., 1998; Bernardi et al., 2015).

Several studies have undertaken analyses of nicotine-induced alterations in the brain proteome. These studies include investigations of whole brain tissue (Miller et al., 2018; Paulo et al., 2018; Koul et al., 2020) or specific brain regions, including hippocampus (Matsuura et al., 2016; Zhu et al., 2017), cortex (Matsuura et al., 2016; Hwang and Li, 2006; McClure-Begley et al., 2016), amygdala (Hwang and Li, 2006), dorsal striatum (Petruzziello et al., 2013; Hishimoto et al., 2016; Hwang and Li, 2006; Yeom et al., 2005), NAc, and VTA (Hwang and Li, 2006) in mice and rats. Nicotine administration ranged from an acute injection 48 hours prior to tissue collection (Hishimoto et al., 2016) to 6 months of nicotine administration through drinking water (Matsuura et al., 2016). Despite evidence for significant sex differences in nicotine addiction, 7 of the 10 studies published to date used only male animal subjects, one study only female animal subjects, and one study did not report the sex of its animal subjects. Only one study used both male and female animal subjects and specifically focused on the proteomic signatures of brain-derived extracellular vesicles in their analysis of sex differences after long-term nicotine self-administration (Koul et al., 2020).

In this study, we used male and female mice to investigate baseline sex differences in the proteome of the mesolimbic system, specifically the VTA and NAc shell, as well as changes in response to a sub-chronic, rewarding regimen of nicotine administration and a chronic regimen followed by withdrawal. To quantify changes in protein abundance, we used isobaric labeling with a TMT 10-plex system, off-line fractionation, and tandem mass spectrometry. This approach results in less variation between runs, more efficient analysis due to sample multiplexing, and improved depth and signal-to-noise ratio (Rauniyar and Yates, 2014; Mansuri et al., 2020). In order to determine the effect of nicotine and of sex, we conducted pairwise analyses of (1) males or females receiving control treatment, (2) males receiving control or nicotine treatment, and (3) females receiving control or nicotine treatment. Further, we explored functional implications of the differentially expressed proteins through pathway enrichment analyses and determined which proteins were significantly altered across experiments to identify converging effects of sex and/or nicotine.

## Materials and Methods

### Animals

Male and female C3H/HeJ (sub-chronic nicotine) and C57Bl/6J (chronic nicotine and withdrawal) mice were obtained from The Jackson Laboratory (Bar Harbor, ME, United States) at 9–11 weeks of age. Nicotine or control treatments began at 10–12 weeks of age, following at least one week of acclimation to the vivarium. Mice were group-housed with cagemates of the same sex and strain, maintained on a 12-h light-dark cycle (lights on at 7:00 AM), and provided standard chow. All procedures were approved by the Yale University Institutional Animal Care and Use Committee.

### Sub-Chronic Nicotine Exposure

Sub-chronic nicotine administration was used in the setting of conditioned place preference (CPP) training. Due to the variability in effective CPP conditioning paradigms, we used C3H/HeJ mice and a training schedule which has successfully produced CPP in both male and female mice in our hands (Lee et al., 2020) to ensure the behavioral relevance of the nicotine administration paradigm. Briefly, mice were handled for 3 days, with gentle stroking of the back and scruff of the neck on the first day, light scruffing of the neck on the second day, and then scruff while pressing syringe (without needles) to back of neck on the third day to simulate a subcutaneous (s.c.) injection. On day 4, animals were given a baseline chamber preference test, where they freely explored the three chambers of the apparatus for 15 min and the time spent in each chamber was recorded. On days 5–10, mice were trained once a day with either saline (10 ml/kg, s.c.) or nicotine injections (nicotine ditartrate salt, NIDA Drug Supply Program; free base concentrations of 0.5 mg/kg for males and 0.75 mg/kg for females in saline at 10 ml/kg, s.c.) preceding a 30-min session in one of the two end chambers. On alternate days, mice received the alternate injection prior to a 30-min session in the alternate chamber. Different doses of nicotine were used to produce similar levels of nicotine reward in male and female mice, accounting for sex differences in nicotine response and sensitivity, including pharmacokinetic differences in nicotine absorption and metabolism (Kota et al., 2007, 2008; Lenoir et al., 2015; Pogun and Yararbas, 2009). We have previously demonstrated that the specific doses used in this study produce CPP in C3H/HeJ mice (Lee et al., 2020). The nicotine group received alternating injections of nicotine and saline on days 5–10, while the control group received saline on all days while alternating chambers on days 5–10. On day 11, mice were again tested for chamber preference in a 15-min session and sacrificed 10–30 min after preference testing.

### Chronic Nicotine Exposure and Withdrawal

C57Bl/6J mice were exposed to nicotine chronically in the drinking water as described (Jung et al., 2016), and brain samples were collected approximately 24 h after the nicotine was withdrawn. This schedule and mode of nicotine administration has been used in C57BL/6J mice to produce nicotine-induced neuronal adaptations (Brunzell et al., 2003; Caldarone et al., 2008; Paulo et al., 2018). Further, the withdrawal period is sufficient to produce physiologic and behavioral symptoms of withdrawal in mice, including molecular adaptations in the mesolimbic system, somatic signs, and affective signs of withdrawal (Jackson and Imad Damaj, 2013; Damaj et al., 2003; Locklear et al., 2012). Nicotine ditartrate salt (NIDA Drug Supply Program) was dissolved in 2% saccharin drinking water to a final free base nicotine concentration of 200 μg/ml. The corresponding control group received 0.2% tartaric acid (Sigma-Aldrich, St. Louis, MO, United States) in 2% saccharin drinking water, which was matched in pH to the nicotine solution (pH 3.7–3.8). Nicotine or saccharin control solutions were the only available sources of drinking water for 21 consecutive days. Treatment drinking water was replaced with normal drinking water following the 21-day treatment period, and tissue was collected 24 h after this switch.

### Tissue Collection

Mice were euthanized by rapid decapitation and brains were quickly removed from the skull and placed on a brain matrix to create 1 mm coronal sections from the frontal cortex through the midbrain. These coronal sections were then carefully placed into a petri dish of cold 1× PBS over ice. Bilateral 1 mm punches were taken from NAc shell- and VTA-enriched regions, deposited into separate 1.5 ml Eppendorf tubes, and immediately frozen on dry ice.

Four mice from each group (sex × treatment) in each experiment (sub-chronic or chronic nicotine administration) were included for proteomic analysis. Samples were excluded if there were any technical difficulties in collecting a sufficient amount of tissue using the brain punch.

### Proteomics Sample Preparation by Protein Extraction, Digestion, Labeling, and Pooling

**FIGURE 1:**
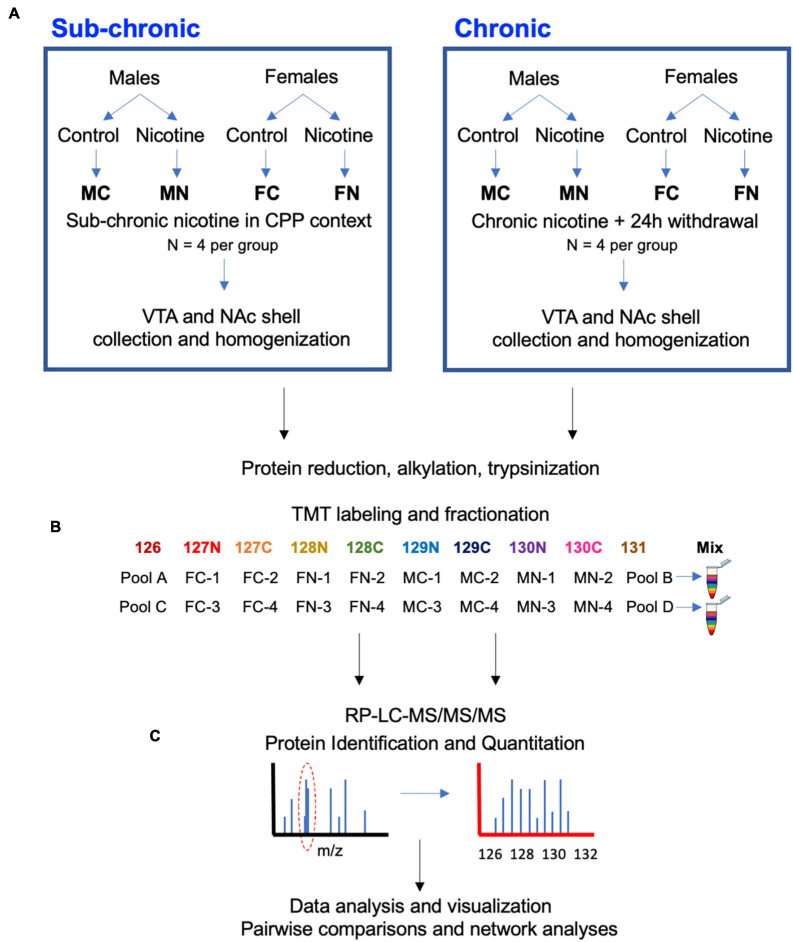
Experimental design and workflow. **(A)** Male and female mice were treated with nicotine or control solutions. In C3H/HeJ mice, sub-chronic nicotine or saline was administered subcutaneously on alternating days for a total of 6 days in a CPP testing paradigm (*n* = 4 per group). In C567BL/6J mice, nicotine or saccharin was administered in drinking water chronically for 21 days, followed by a 24-h withdrawal period (*n* = 4 per group). About 10–30 min after CPP testing in C3H/HeJ mice, or 24 h after cessation of nicotine or saccharin treatment in C57BL/6J mice, subjects were sacrificed and the VTA and NAc shell were collected from each brain by 1 mm punch biopsy on 1 mm-thick coronal sections. **(B)** Tissue samples were subsequently processed for proteomic analysis with a TMT10-plex isobaric labeling strategy. **(C)** Data were quantified, normalized, analyzed, and visualized using MaxQuant and Perseus software and the online STRING database tool.

Mouse tissue punches were lysed in fresh lysis buffer [RIPA buffer, 0.5% sodium deoxycholate, containing 1× PhosSTOP phosphatase inhibitor (Roche) and 1× cOmplete protease inhibitor cocktail (Roche)]. The homogenate was incubated at 4°C on ice for 10 min and then sonicated on ice for 3 × 10 s with 30 s intervals. A soluble fraction was obtained following centrifugation for 10 min at 14,000 × *g* at 4°C (Tan et al., 2017). A BCA protein concentration assay (Thermo Fisher Scientific) was performed and confirmed by running a short SDS gel with BSA standards stained with Coomassie blue. Protein (50 μg) for each sample then underwent acetone precipitation, reduction, alkylation, and trypsin (Promega, 1:50 w/w) digestion overnight at 37°C, then quenching with 1% trifluoroacetic acid. RP C18 cartridges (The Nest Group, Southborough, MA, United States) were used to desalt peptides, after which samples were dried with speed-vac. Samples were resuspended in 50 mM HEPES (pH 8.5) and labeled with TMT 10-plex reagents according to the manufacturer’s instructions (Thermo Fisher Scientific; see also Figure 1). A label efficiency test was performed on 1/20th of each sample prior to equally mixing the 10 channels (8 samples and 2 pooled controls generated by mixing equal amount of proteins from all 8 samples used in each run), then samples were dried prior to fractionation and analysis.

### High-pH Reversed-Phase Fractionation and SPS-MS3 TMT Data Acquisition

Fractionation and TMT data acquisition were performed as previously described (Mervosh et al., 2018). Briefly, high-pH reversed-phase C18 peptide fractionation was performed on an ACQUITY UPLC H-class system (Waters Corporation, Milford, MA, United States) equipped with an ACQUITY UPLC BEH C18 column (1.7 μm, 2.1 mm × 50 mm). Elution was performed as previously described (Mervosh et al., 2018), with a flowrate of 0.3 ml/min and gradient of 2% of mobile phase A (10 mM ammonium acetate, pH 10) to 37% mobile phase B (10 mM ammonium acetate in 90% acetonitrile, pH 10) in 19.8 min. In total 60 fractions were collected, pooled into five fractions, dried, and reconstituted in loading buffer (0.2% trifluoroacetic acid, 2% acetonitrile in water).

Reversed phase-liquid chromatography-mass spectroscopy (RP-LC-MS/MS/MS) was performed at the Yale/Keck MS & Proteomics Resource with a nanoACQUITY UPLC system (Waters Corporation, Milford, MA, United States) connected to an Orbitrap Fusion Tribrid (Thermo Fisher Scientific, San Jose, CA, United States) as described (Mervosh et al., 2018). SPS-MS3 scanning was performed as described previously (Mervosh et al., 2018), except that the maximum injection time was set to 60 ms, and dynamic exclusion was enabled for a duration of 30 s for the full scan. CID-MS fragmentation isolation mode was set to quadrupole and isolation width was set to 1.6 m/z. MS3 scans were produced with higher-energy collision dissociation (HCD) for the top 10 fragment ions for each peptide MS2 and were analyzed in the Orbitrap at a resolution of 60,000. The maximum injection time was set to 120 ms and automatic gain control (AGC) target value was set to 1 × 10^5^.

### Quantification and Normalization of Peptides and Proteins

Raw files were processed with MaxQuant (MaxQuant, RRID:SCR_014485) version 1.6.1.0 with a false-discovery rate (FDR) < 0.01 at the level of proteins, peptides and modifications using the Andromeda search engine integrated into the MaxQuant environment, and using the Mouse UniProt FASTA database (16916 sequences available, retrieved October 2018, UniProtKB, RRID:SCR_004426). For Group Specific Parameter changes, “type” was set as “reporter ion MS3” and “10plex TMT.” Oxidized methionine (M) and acetylation (protein N-term) were selected as variable modifications, and carbamidomethyl (C) as a fixed modification with minimum peptide length of seven amino acids. Trypsin was selected as the protease allowing for up to two missed cleavages, and the peptide mass was limited to a maximum of 4,600 Da. Quantification of peptides and proteins was performed by MaxQuant using “unique + razor peptides” including unmodified peptides and modified peptides [oxidation (M) and Protein N-term acetyl]. “Match between runs” (MBR) was enabled with a matching time window of 0.7 min. In general, values of parameters in MaxQuant have not been changed from their default values unless explicitly stated (Sowers et al., 2019).

These search results were exported as text files, along with their TMT reporter ion intensities (Supplementary Table 1). Three normalization steps were implemented as described (Plubell et al., 2017; Robinson and Oshlack, 2010) using in-house R scripts in RStudio (RStudio, RRID:SCR_000432) version 1.3.1093. Each experiment (i.e., sub-chronic and chronic nicotine administrations) consisted of two TMT 10-plex runs with two biological replicates of each experimental group (sex × treatment) and two pooled channels of equal ratios (i.e., equal amount of proteins) of the eight experimental channels within the TMT run. First, data were normalized within each TMT 10-plex experiment to account for small variations in sampling loading and labeling efficiency. To accomplish this first normalization, a global scaling factor was applied to the total ion reporter intensity of each channel such that each channel was adjusted to the average total intensity across all ten channels. A second normalization step called “trimmed means of M values” (TMM), originally developed for RNA-seq data (Robinson and Oshlack, 2010), was used to align the centers of intensity distributions across samples within each TMT experiment. This provided robust normalization across samples, which can skew detection of differentially expressed proteins (Robinson and Oshlack, 2010; Branson and Freitas, 2016). Third, the data were normalized across TMT experiments (batch correction) using “internal reference scaling” (IRS; Plubell et al., 2017; Mansuri et al., 2020) for chronic VTA or Limma (Brusniak et al., 2008; Margolin et al., 2009; Ting et al., 2009; Jankova et al., 2011; Schwammle et al., 2013; Zhao et al., 2013) for sub-chronic VTA, sub-chronic NAc, and chronic NAc due to higher variability of data in the latter datasets. IRS uses reporter ion intensities from the two pooled sample reference channels within each TMT experiment to create scaling factors for each protein in the eight experimental channels. All normalized data are available in Supplementary Table 2.

### Statistical Analysis

Normalized ion intensities were statistically analyzed in Perseus software (Perseus, RRID:SCR_015753) version 1.6.0.7. Values were first log2 transformed to achieve normal distribution. Proteins were filtered to remove “only identified by site,” “reverse,” and “potential contaminants.” In addition, proteins with missing values in any individual sample were filtered out. For the sub-chronic VTA, sub-chronic NAc, and chronic NAc analyses, an additional step of multiple ANOVA tests with FDR < 0.05 was implemented due to the relatively lower numbers of proteins identified in these experiments (<2,000) compared to the chronic VTA experiment (>3,000), and the higher variability in protein expression values observed in those three analyses. This step reduced the variability in protein expression values in the sub-chronic VTA, sub-chronic NAc and chronic NAc data, thereby improving data quality as assessed by the clustering of replicates in principle component analyses (PCA). Pairwise comparisons between male control and female control, male nicotine (MN) and male control (MC), and female nicotine (FN) and female control (FC) in each experiment were then conducted to determine differentially regulated proteins by sex and by nicotine treatment. In each pairwise comparison above, the second group served as the reference group (female control, male control and female control, respectively). Moderated *t*-tests were performed with FDR < 0.05 and s0 = 0.5. The s0 parameter incorporates expression values into significance determinations, such that s0 = 0 relies solely on FDR to determine significance and s0 > 0 uses both fold change and FDR cutoffs. Default settings for FDR determination were used (permutation-based FDR with 250 randomizations applied).

### Bioinformatics Analysis

Pathway enrichment analyses were conducted using the Search Tool for the Retrieval of Interacting Genes (STRING) database (STRING, RRID:SCR_005223) version 11.0^2^ (Szklarczyk et al., 2019). Significantly regulated proteins, as determined by Perseus in pairwise comparisons, were submitted without expression values to the STRING database for gene ontology (GO) and pathway enrichment analyses.

Further sub-network analyses were performed by importing protein interaction data into Cytoscape (Cytoscape, RRID:SCR_003032) version 3.8.2. The required interaction score and FDR were set at minimums of 0.7 and 1%, respectively. The Molecular Complex Detection (MCODE) plug-in (MCODE, RRID:SCR_015828) (Bader and Hogue, 2003) was used in Cytoscape with cut off-values of node score = 0.2, degree = 4, k-core = 4, and maximum depth = 100 to identify protein–protein interaction (PPI) networks within the data. The proteins in each network cluster were further submitted to the DAVID database (DAVID, RRID:SCR_001881) for GO and KEGG pathway enrichment analysis.

Raw data and results of analyses are provided in Supplementary Tables 1–8, as summarized in Table 1.

**TABLE 1:** Summary of data provided in Supplementary Tables 1–8.

Table #	Table File Name	Description of contents
S1	Supplementary Table 1_All-Unnormalized	Non-normalized intensity values for all proteins identified by mass spectrometry. Data are shown for 4 biological replicates of each experimental group (sex × treatment) and 4 pooled reference samples. Each of the 4 sheets shows this data for one brain region (VTA or NAc) after one nicotine administration experiment (sub-chronic or chronic).
S2	Supplementary Table 2_All-Normalized	Normalized intensity values for all proteins identified by mass spectrometry. Data are shown for 4 biological replicates of each experimental group (sex × treatment) and 4 pooled reference samples. Each of the 4 sheets shows this data for one brain region (VTA or NAc) after one nicotine administration experiment (sub-chronic or chronic).
S3	Supplementary Table 3_Pairwise Sub-chronic VTA	The results of pairwise comparisons to determine which proteins were differentially expressed between MC vs. FC, FN vs. FC, and MN vs. MC in the VTA after sub-chronic nicotine administration.
S4	Supplementary Table 4_Pairwise Sub-chronic NAc	The results of pairwise comparisons to determine which proteins were differentially expressed between MC vs. FC, FN vs. FC, and MN vs. MC in the NAc after sub-chronic nicotine administration.
S5	Supplementary Table 5_Pairwise Chronic VTA	The results of pairwise comparisons to determine which proteins were differentially expressed between MC vs. FC, FN vs. FC, and MN vs. MC in the VTA after chronic nicotine administration.
S6	Supplementary Table 6_Pairwise Chronic NAc	The results of pairwise comparisons to determine which proteins were differentially expressed between MC vs. FC, FN vs. FC, and MN vs. MC in the NAc after chronic nicotine administration.
S7	Supplementary Table 7_Commonly altered proteins	Proteins that were found to be differentially expressed in multiple pairwise comparisons. Each of the 4 sheets shows these commonly altered proteins for one type of pairwise comparison (e.g., MC vs. FC) in one brain region (VTA or NAc) across strains or nicotine administration experiments.
S8	Supplementary Table 8_Top 5 KEGG Pathways	Five most significantly enriched KEGG pathways for differentially expressed proteins in each pairwise comparison. Each of the 3 sheets shows the KEGG pathways enriched in each pairwise comparison (MC vs. FC, FN vs. FC, MN vs. MC) in one brain region (VTA or NAc) in one strain or nicotine administration experiment.

## Results

### Quantitative Proteomic Analysis of VTA and NAc Shell After Sub-Chronic and Chronic Administration of Nicotine

The VTA or NAc shell proteomes were examined after sub-chronic nicotine administration and after withdrawal from chronic nicotine administration as shown by the workflow depicted in Figure 1. Sub-chronic nicotine was administered at doses and on a schedule that induces CPP in C3H/HeJ mice (Lee et al., 2020). Chronic nicotine was administered to C57BL/6J mice through drinking water for 21 days, followed by 24 h of normal drinking water administration prior to tissue collection, at which point mice can exhibit signs of withdrawal (Jackson and Imad Damaj, 2013; Damaj et al., 2003). Tissue was then processed, TMT 10-plex isobaric tags were applied to biological replicates of the experimental groups and pooled reference samples, and each set of labeled samples was pooled, fractionated and run through LC-MS/MS/MS (Figure 1).

Proteins were identified by applying the criteria of <1% FDR for peptides and proteins, and no missing value across the biological replicates in sex × treatment groups in each brain region after each nicotine treatment. These criteria identified 1,813 proteins in the VTA (Supplementary Table 1A) and 1,562 proteins in the NAc shell (Supplementary Table 1B) in the sub-chronic nicotine groups. After chronic nicotine exposure and withdrawal, there were 3,215 proteins identified and quantified in the VTA (Supplementary Table 1C) and 1,827 in the NAc shell (Supplementary Table 1D). Data were normalized to account for within, and across, mass spectrometry analyses (see section “Materials and Methods” and Supplementary Tables 2A–D). In order to facilitate more meaningful pairwise comparisons between sex and treatment groups, an additional multiple ANOVA step was implemented prior to differential protein expression analysis for VTA sub-chronic, NAc sub-chronic, and NAc chronic data sets. In these data sets, the multiple ANOVA step helped address the lower numbers of proteins identified (<2,000) and higher variability in protein quantitation that was found. Following the multiple ANOVAs, the proteins submitted for differential protein expression analysis were 286 for VTA sub-chronic, 404 for NAc sub-chronic, and 235 for NAc chronic.

**FIGURE 2:**
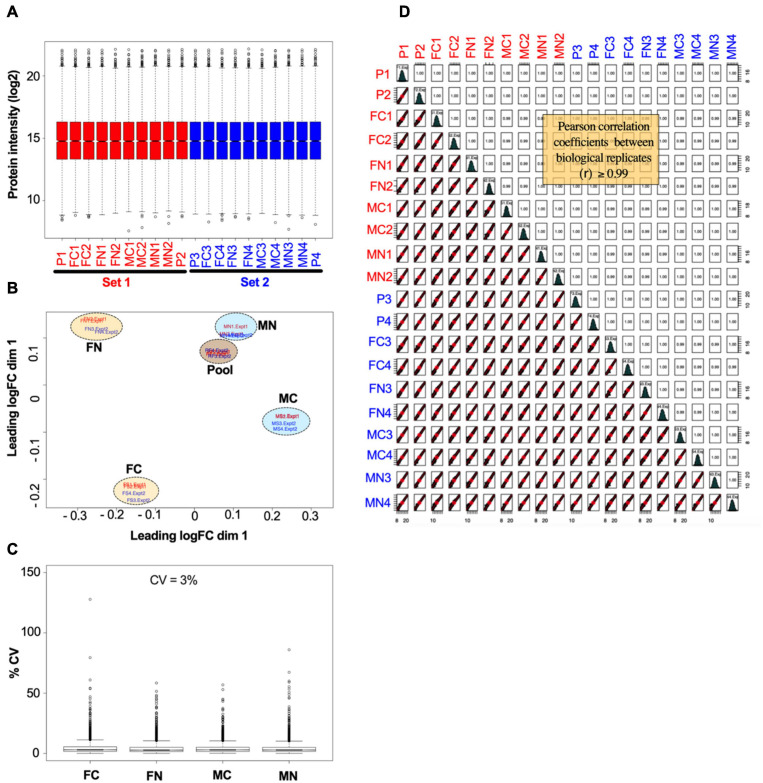
Example of data normalization by sample loading, TMM, and IRS, using data from the VTA after chronic nicotine and withdrawal. **(A)** Normalized intensity values for each reference pool (P1, P2, P3, P4) and experimental sample in each of the two TMT experimental sets (red and blue) are shown as box plots. **(B)** Principal component analysis of sample intensity values shows clustering of the samples by experimental groups and reference pooled samples, where each oval encompasses the biological replicates run in each TMT experimental set. **(C)** The coefficients of variance of each experimental group averaged ∼3% after normalization. **(D)** Quality control analysis showing, for each biological replicate, scatter plots of normalized quantification values (log_2_ of TMT intensities) and Pearson correlation coefficients, which ranged from 0.99 to 1.00.

The reproducibility and overall quality of the data were confirmed. Figure 2 shows normalized data from the VTA after chronic nicotine administration and withdrawal. The distribution of individual protein intensity values after normalization were highly similar between individual biological samples, as illustrated by box plots (Figure 2A). A principal component analysis (PCA) indicates more variability between experimental groups than within groups, as illustrated by the clustering of biological replicates when mapped across the first two dimensions of the PCA (Figure 2B). The coefficients of variance were low across groups, averaging 3% (Figure 2C). Finally, multiple scatter plots of individual proteins between biological replicates demonstrated Pearson correlation coefficients ≥0.99 (Figure 2D). Overall, these analyses indicate the high quality of the data.

### Pairwise Comparisons of Differentially Regulated Proteins by Sex and Treatment Group

**FIGURE 3:**
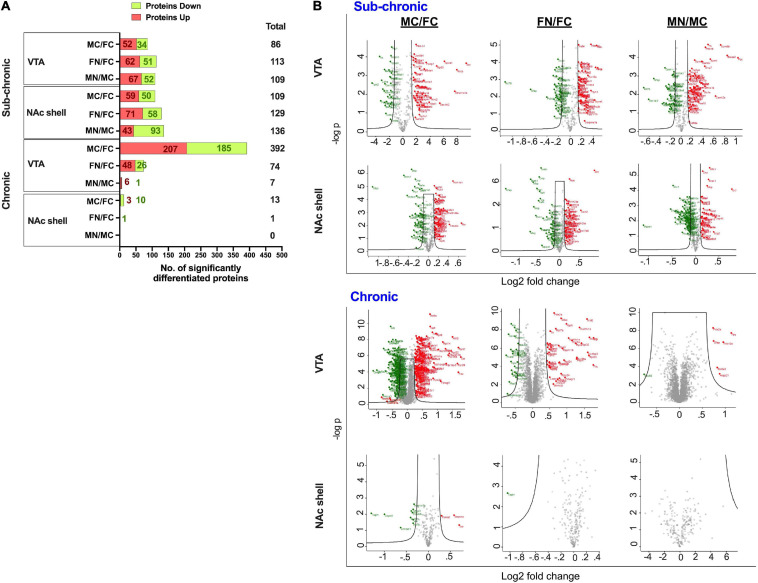
Significantly altered proteins in pairwise comparisons between male vs. female and nicotine vs. control groups. **(A)** Summary of differentially regulated proteins in each pairwise comparison. **(B)** Volcano plots of log_2_ fold change and –log_10_ *p*-values are shown for each pairwise comparison in VTA and NAc shell after sub-chronic or chronic nicotine administration in C3H/HeJ or C57BL/6J mice. Black lines within the graph show the significance cutoffs as determined in Perseus with s0 = 0.5 and FDR < 0.05. Proteins meeting significance criteria are colored. Red indicates an increase in protein abundance, and green indicates a decrease in protein abundance.

Differentially regulated proteins in each group were calculated as described in the section “Materials and Methods,” such that moderated *t*-tests were performed with FDR < 0.05 and s0 = 0.5. This analysis is depicted as summary data in Figure 3A and in volcano plots in Figure 3B. The analysis included pairwise comparisons of differentially regulated proteins in either VTA or NAc shell between male control and female control (MC vs. FC) samples, female nicotine and female control groups (FN vs. FC), and male nicotine and male control groups (MN vs. MC). After sub-chronic nicotine administration in the VTA of C3H/HeJ mice, slightly fewer proteins were differentially expressed between MC vs. FC (86 proteins) compared to the proteins differentially expressed in FN vs. FC (113 proteins) and MN vs. MC (109 proteins) comparisons (Figure 3). A similar pattern in numbers of differentially regulated proteins was observed in the NAc shell after sub-chronic nicotine administration, with 109, 129, and 136, proteins significantly altered in MC vs. FC, FN vs. FC, and MN vs. MC mice, respectively (Figure 3).

In contrast, there were more strikingly varied patterns of differential protein expression in C57BL/6J mice. In the VTA, 392 proteins were differentially expressed between MC vs. FC, far exceeding the 74 proteins that were differentially expressed in FN vs. FC mice, and the 7 proteins that were differentially expressed in MN vs. MC mice (Figure 3). An overall similar pattern was observed in the NAc shell, but of a different magnitude. In the NAc shell, 13 proteins were differentially expressed in MC vs. FC, while there was only one protein that was significantly decreased in FN vs. FC mice, and zero proteins that were differentially regulated by nicotine exposure in male mice (Figure 3). The identities of all proteins, as well as their fold change and q-values, are available online (Supplementary Tables 3–6).

### Comparison of Commonly Altered Proteins Across Sex and Treatment Groups

**FIGURE 4:**
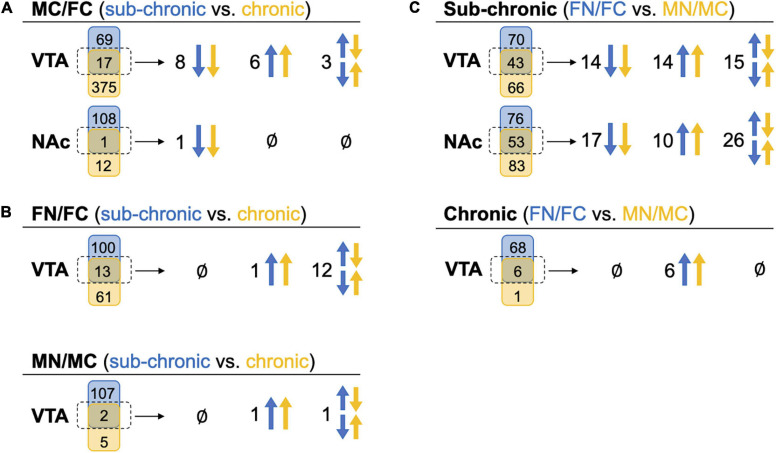
Comparison of commonly altered proteins across sex and treatment groups. Headings indicate the pairwise tests being compared, while in parentheses the blue and yellow text indicate the groups from which those pairwise tests are being drawn for comparison. Under each heading, a Venn diagram for each brain region shows the numbers of proteins that were uniquely (blue or yellow shape) or commonly (middle green shape) identified as significantly altered proteins in these comparisons. The commonly altered proteins, outlined in a dotted line, are further delineated to be commonly up-regulated in both pairwise tests across blue and yellow groups (both arrows up), commonly down-regulated (both arrows down), or commonly identified but altered in different directions (set of arrows in opposite directions). **(A)** Significantly altered proteins in MC vs. FC for each brain region compared across the C3H/HeJ and C57BL/6J mice used for the sub-chronic and chronic nicotine administration groups, respectively. **(B)** Significantly altered proteins after nicotine vs. control treatment in each sex compared across nicotine administration groups. NAc is not shown because there were no commonly altered proteins across nicotine administration groups. **(C)** Significantly altered proteins after sub-chronic or chronic nicotine administration compared across sex.

We further evaluated the pairwise comparisons to identify similarities in sub-chronic vs chronic nicotine-induced and sex-dependent alterations in protein expression within the NAc shell and the VTA (Figure 4). Differential expression of the same proteins in multiple comparisons provides convergent evidence of the regulation of those proteins. For example, if the same set of proteins are differentially expressed in MC vs. FC in both C3H/HeJ and C57BL/6J mice, it would strengthen the suggestion that expression of those proteins is sex-dependent at baseline. In the VTA in the absence of nicotine exposure, 17 proteins were commonly differentially expressed between sexes, representing 19.8% (17/86) and 4.3% (17/392) of the total proteins differentially expressed between MC vs. FC in the VTA of C3H/HeJ mice and C57BL/6J mice, respectively (Figure 4A and Supplementary Table 7A). 14 of the 17 (82.4%) overlapping proteins were differentially expressed in the same direction, including Ppp1r1b [dopamine and cAMP-regulated phosphoprotein of 32 kDa (DARPP-32)] which was expressed at higher levels in males. In the NAc shell, only one protein, Krt76, was commonly regulated in C3H/HeJ (0.9%, or 1 out of 109 total differentially regulated proteins) and C57BL/6J (7.7% or 1/13) MC vs. FC comparisons (Figure 4A and Supplementary Table 7B).

For each sex, the proteins regulated by sub-chronic nicotine and chronic nicotine were compared to find proteins that may be key regulators of nicotine’s effects across reward and withdrawal (Figure 4B, Supplementary Table 7). In the VTA FN vs. FC comparisons, there were 13 proteins in common between sub-chronic (11.5% or 13 of 113 total differentially regulated proteins) and chronic (17.6% or 13/74) nicotine administration groups. Of those 13 proteins, only Ppp1r1b was altered in the same direction (increased protein levels following nicotine exposure; Figure 4B and Supplementary Table 7C). In the VTA MN vs. MC comparisons, there were two overlapping proteins [glial fibrillary acidic protein (GFAP) and Ppp1r1b], or 1.8% (2/109) and 28.6% (2/7) of differentially regulated proteins in sub-chronic and chronic nicotine groups, respectively (Figure 4B and Supplementary Table 7D). Similar to the FN vs. FC comparisons, only Ppp1r1b was increased in both sets of male VTA comparisons (Figure 4A). Notably, these similarities in protein alterations are within the same brain region and same pairwise comparisons of sex and treatment groups, but the tissue are from different mouse strains with different nicotine treatment protocols (i.e., C3H/HeJ for sub-chronic administration, and C57BL/6J for chronic administration and withdrawal).

There was only one protein significantly altered in the NAc shell in FN vs. FC after chronic nicotine administration and withdrawal, and none in MN vs. MC. This protein, Tagln, was altered in opposite directions in sub-chronic and chronic groups.

Within sub-chronic and chronic nicotine administration experiments, nicotine-induced protein alterations between sexes were compared to find sex-independent effects of nicotine (Figure 4C). Between FN vs. FC and MN vs. MC comparisons in the VTA after sub-chronic nicotine administration, there were 43 proteins commonly identified as differentially regulated proteins representing 38.1% (43/113) and 39.4% (43/109) of proteins with nicotine-induced alterations in expression in female and male mice, respectively (Figure 4C and Supplementary Table 3). 65.1% (28/43) of the commonly regulated proteins were altered in the same direction, including GFAP, an astrocyte marker which was downregulated by sub-chronic nicotine in both males and females, and Ppp1r1b which was up-regulated in both sexes. DOPA decarboxylase (DDC) and tyrosine hydroxylase (TH), two proteins involved in dopamine synthesis, were commonly regulated but in different directions: both were decreased by sub-chronic nicotine in the female VTA, but increased in male VTA (Supplementary Table 3). In the NAc after sub-chronic nicotine administration, 41.1% (53/129) of proteins differentially expressed in FN vs. FC were also altered by nicotine in MN vs. MC, or 39.0% (53/136) of proteins in male mice (Figure 4C and Supplementary Table 4). 50.9% (27/53) of the commonly altered proteins were also altered in the same direction, including glutamate decarboxylase 2 (Gad2) and vesicular glutamate transporter 2 (Slc17a6), which were up-regulated by nicotine in both males and females, and potassium/sodium hyperpolarization-activated cyclic nucleotide-gated channel 1 (Hcn1) which was downregulated by nicotine in both male and female NAc. Examples of proteins commonly identified but altered in different directions include monoamine oxidase B (Maob), which was increased by sub-chronic nicotine in female NAc but decreased in male NAc, and proenkephalin-A (Penk), which was decreased by sub-chronic nicotine in female NAc but increased in male NAc (Supplementary Table 4).

In the VTA after chronic nicotine administration and withdrawal, 6/7 (85.7%) proteins significantly up-regulated in MN vs. MC were also up-regulated in FN vs. FC (8.1%, 6/74; Figure 4C). These proteins include GFAP, a marker of astrocytes, and Ppp1r1b (DARPP-32), as well as regulator of calmodulin signaling (RCS or Arpp21), sodium-dependent dopamine transporter (DAT; Slc6a3), and two phosphodiesterases, Pde10a and Pde2a (Supplementary Table 5).

### Pathway Enrichment Analyses of Differentially Regulated Proteins in Sex and Treatment Comparisons

**FIGURE 5:**
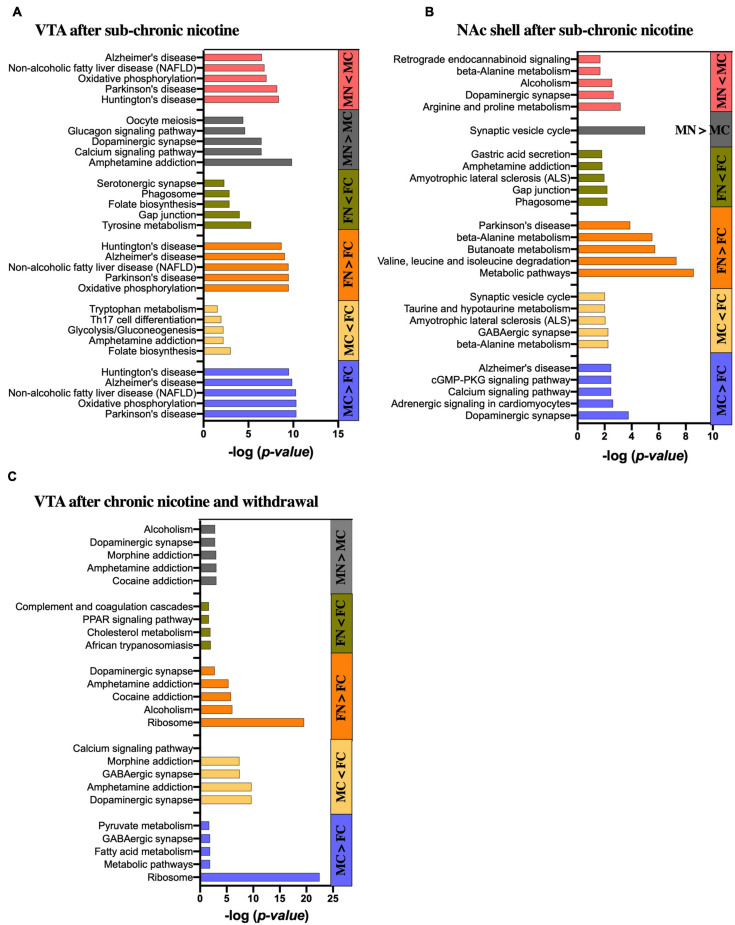
KEGG pathway analysis of significantly altered proteins in each pairwise comparison by sex and treatment group. Bar graphs show the five most significantly enriched KEGG pathways in the significantly up- and down-regulated proteins of each pairwise comparison as indicated in the **(A)** VTA after sub-chronic nicotine administration, **(B)** NAc shell after sub-chronic nicotine administration, and **(C)** VTA after chronic nicotine administration and withdrawal. Where less than five KEGG pathways are shown, only that number of pathways was identified as significantly enriched.

The STRING database was used to identify emergent patterns in the proteins that were differentially expressed within pairwise comparisons. The STRING database analysis displays functional enrichment by GO categories, including biological process, molecular function and cellular component, as well as KEGG pathways, multiple protein domain databases, and other enrichment categorizations. The five most significantly enriched KEGG pathways for the different comparisons are shown in Figure 5 to focus on possible functional implications of the significantly altered proteins. Significantly altered proteins that are members of the gene sets for the top five enriched KEGG pathways in each pairwise comparison are shown in Supplementary Table 8.

#### STRING Analysis of Differentially Regulated Proteins in VTA After Sub-Chronic Nicotine Administration

Differentially expressed proteins between the male and female control groups in the VTA of C3H/HeJ mice were submitted for STRING analysis. For the 52 proteins with significantly greater expression in the VTA of MC vs. FC C3H/HeJ mice, the five most significantly enriched KEGG pathways out of 14 total were Parkinson’s disease, oxidative phosphorylation, non-alcoholic fatty liver disease, Alzheimer’s disease and Huntington’s disease (Figure 5A and Supplementary Table 8A). For the 34 proteins with significantly greater expression in FC vs. MC mice, the five most significantly enriched KEGG pathways of seven total included folate biosynthesis, amphetamine addiction, glycolysis/gluconeogenesis, Th17 cell differentiation, and tryptophan metabolism (Figure 5A and Supplementary Table 8A).

In the VTA after sub-chronic nicotine administration, the five most significantly enriched KEGG pathways of the 22 total from the 62 proteins upregulated in FN vs. FC were composed of the same set of proteins (Cox6b1, Cyc1, Ndufa6, Ndufa9, Ndufb10, Ndufs3, Ndufs5, Ndufs6, Ndufv2, and mt-Co2), and were the same five pathways as those enriched for proteins upregulated in MC over FC (Figure 5A and Supplementary Table 8A). For the 51 proteins decreased in FN vs. FC, nine KEGG pathways were significantly enriched in total, including several terms related to dopamine synthesis, metabolism or signaling such as tyrosine metabolism, dopaminergic synapse, amphetamine addiction, and phenylalanine metabolism (Figure 5A and Supplementary Table 8A).

Sixty-seven proteins significantly upregulated in MN vs. MC were submitted to the STRING database, and the five most significantly enriched KEGG pathways terms of 46 total in these proteins included amphetamine addiction, calcium signaling, and dopaminergic synapse (Figure 5A). The list also included glucagon signaling and oocyte meiosis, with four of the six genes representing each of those gene sets also included in the gene sets for amphetamine addiction, calcium signaling and dopaminergic synapse terms (Camk2a, Camk2b, Camk2g, and Ppp3ca). For the 52 proteins that were decreased in MN vs. MC, the five most significantly enriched KEGG pathways of 14 total included the same five most significantly enriched pathways that were upregulated in MC vs. FC and in FN vs. FC, although the proteins included did not overlap completely (Figure 5A and Supplementary Table 8A).

#### STRING Analysis of Differentially Regulated Proteins in NAc After Sub-Chronic Nicotine Administration

For the 59 proteins with significantly greater expression in the NAc of MC vs. FC C3H/HeJ mice, the five most significantly enriched KEGG pathways included signaling-related terms such as dopaminergic synapse, calcium signaling pathway, and cGMP-PKG signaling pathway (Figure 5B and Supplementary Table 8B). The full list of 24 also included glutamatergic synapse, cAMP signaling pathway, and retrograde endocannabinoid signaling terms, as well as addiction-related terms. For the 50 proteins with significantly greater expression in the FC over MC mice, metabolic terms including beta-alanine metabolism and taurine and hypotaurine metabolism were among the five most significantly enriched KEGG pathways, and butanoate, alanine, aspartate, and glutamate metabolism were among the total 9 significantly enriched KEGG pathway terms. Neuron-specific KEGG pathway terms that were significantly enriched in FC over MC mice included GABAergic synapse, ALS, and synaptic vesicle cycle (Figure 5B and Supplementary Table 8B).

The five most significantly enriched KEGG pathways among the 71 proteins upregulated in FN vs. FC mice in the NAc shell after sub-chronic nicotine administration included primarily metabolic pathways and one KEGG pathway related to Parkinson’s disease (Figure 5B and Supplementary Table 8B). The full list of 30 pathways included more metabolic pathways, as well as several pathways related to dopaminergic signaling, including dopaminergic synapse, alcoholism, cocaine addiction, amphetamine addiction, and tyrosine metabolism. The five most significantly enriched KEGG pathways of 11 total among the 58 proteins decreased in FN vs. FC included the phagosome, gap junction, ALS, amphetamine addiction, and gastric acid secretion (Figure 5B and Supplementary Table 8B).

There was only one significantly enriched KEGG pathway among the 43 proteins upregulated in MN vs. MC mice in the NAc shell after sub-chronic nicotine: synaptic vesicle cycle. The five most significantly enriched KEGG pathways of 20 total among the 93 proteins decreased in MN vs. MC included terms related to neuropsychiatric functions, such as dopaminergic synapse, alcoholism, and retrograde endocannabinoid signaling, and metabolic terms, including arginine and proline metabolism as one term, and beta-alanine metabolism (Figure 5B and Supplementary Table 8B).

#### STRING Analysis of Differentially Regulated Proteins in VTA After Chronic Nicotine Administration and Withdrawal

For the 207 proteins with greater expression in the VTA of C57BL/6J mice after chronic nicotine administration and withdrawal, the five most significantly enriched KEGG pathways of eight total included ribosome, metabolic pathways, fatty acid metabolism, and pyruvate metabolism, as well as GABAergic synapse (Figure 5C and Supplementary Table 8C). For the 185 proteins with greater expression in the VTA of FC over MC mice, a total of 58 KEGG pathways were significantly enriched. The five most significantly enriched KEGG pathway terms were related to neuronal signaling, including dopaminergic synapse, amphetamine addiction, GABAergic synapse, morphine addiction, and calcium signaling.

Among the 48 proteins upregulated in FN vs. FC mice, the five most significantly enriched KEGG pathways terms of eight total included ribosome, alcoholism, cocaine addiction, amphetamine addiction, and dopaminergic synapse (Figure 5C and Supplementary Table 8C). For the 26 proteins significantly decreased in FN vs. FC mice, there were only four significantly enriched KEGG pathway terms, none of which are specific to neuronal function (Figure 5C and Supplementary Table 8C). For the six proteins significantly increased in MN vs. MC mice, three were directly related to dopaminergic signaling, including Ppp1r1b, Arpp21, and Slc6a3. Accordingly, four of the total six enriched KEGG pathways were related to dopaminergic functions, including cocaine addiction, amphetamine addiction, dopaminergic synapse, and alcoholism (Figure 5C and Supplementary Table 8C). Morphine addiction and purine metabolism were enriched KEGG pathways based on Pde10a and Pde2a proteins matching the representative gene set for those terms.

After chronic nicotine administration and withdrawal, there were no significantly enriched KEGG pathways among the 10 proteins significantly increased in the NAc shell of FC over MC mice.

### Protein–Protein Interaction Network Analysis

**FIGURE 6:**
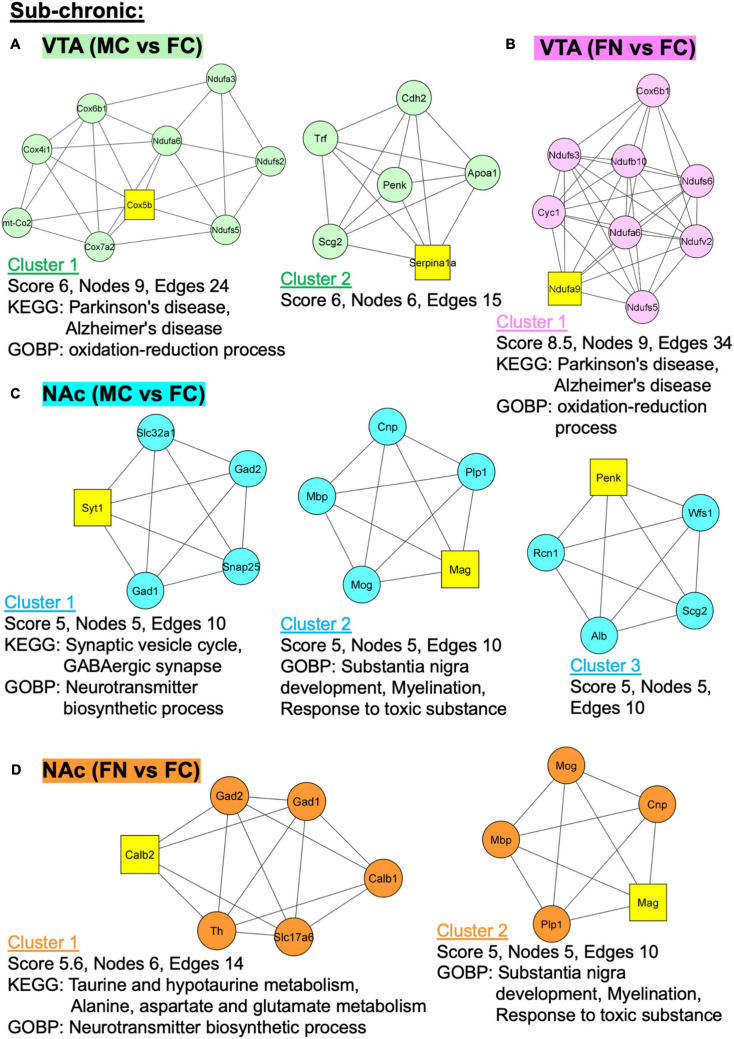
Protein–protein interaction (PPI) network analysis of differentially expressed proteins with and without sub-chronic nicotine. PPI network construction was performed using the STRING database for sex and treatment group comparisons with sufficient numbers of differentially regulated proteins for analysis. Thresholds for analysis were set at an interaction score of >0.7 and FDR of 1%. Further sub-network analysis was performed using the MCODE plug-in in Cytoscape (cut off-values: node score = 0.2, degree = 4, k-core = 4, maximum depth = 100). The top three ranked clusters of networks, or fewer if less than three were identified, are shown. Score, number of nodes, number of edges, and significantly enriched GO and KEGG pathways (*p* < 0.05) as analyzed using DAVID database are shown for each cluster. **(A)** Two clusters were identified for MC vs. FC in the VTA of C3H/HeJ mice used for sub-chronic nicotine administration. **(B)** Only one cluster was identified for FN vs. FC in the VTA after sub-chronic nicotine administration. **(C)** The top three clusters are shown for MC vs. FC in the NAc shell of C3H/HeJ mice used for sub-chronic nicotine administration. **(D)** Two clusters were identified for FN vs. FC in the NAc shell after sub-chronic nicotine administration.

**FIGURE 7:**
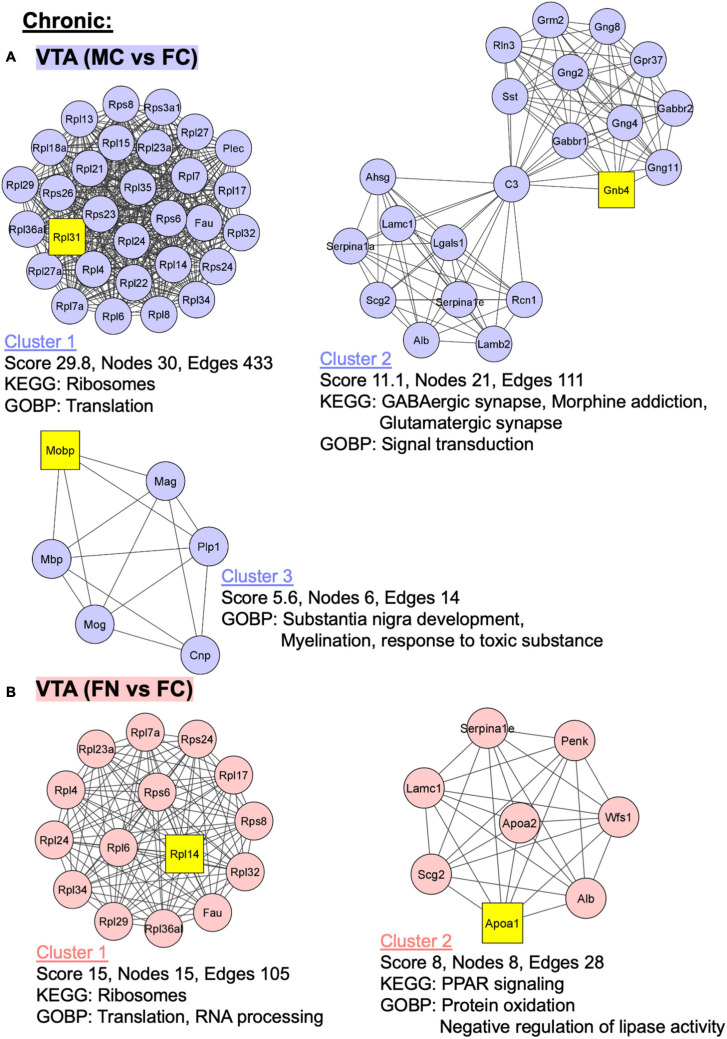
PPI network analysis of differentially expressed proteins with and without chronic nicotine and withdrawal. PPI networks were constructed as described for Figure 6. **(A)** The top three clusters are shown for MC vs. FC in the VTA of C57BL/6J mice used for chronic nicotine administration. **(B)** Two clusters were identified for FN vs. FC in the VTA after chronic nicotine administration and withdrawal.

Additional analyses were performed using MCODE in Cytoscape in order to identify PPI networks. The top three network clusters in each analysis are shown in Figures 6, 7, annotated with the score, number of nodes, and edges in each cluster as well as significantly enriched (*p* < 0.05) GO and KEGG pathways as determined with further DAVID database analysis. The input for these analyses were all significant differentially regulated proteins, both up- and down-regulated, in each pairwise comparison.

In the VTA after sub-chronic nicotine administration, two PPI clusters were identified among proteins differentially regulated in the MC vs. FC comparison (Figure 6A). In cluster 2, Parkinson’s and Alzheimer’s disease KEGG pathways and oxidation-reduction process GO biological process (GOBP) pathways were enriched. In the sub-chronic VTA FN vs. FC comparison, one significant protein network was identified with the same KEGG pathways (Parkinson’s disease, Alzheimer’s disease) and GOBP terms (oxidation-reduction process) enriched as in MC vs. FC (Figure 6B).

In the NAc after sub-chronic nicotine, the top three protein clusters for the MC vs. FC comparisons are shown (Figure 6C). Enriched KEGG pathways among these protein networks include synaptic vesicle cycle and GABAergic synapse, and enriched GOBP pathways include neurotransmitter biosynthetic process, substantia nigra development, myelination, and response to toxic substance. In the sub-chronic NAc FN vs. FC comparison, two PPI network clusters were identified (Figure 6D). Enriched KEGG pathways were taurine and hypotaurine metabolism, and alanine, aspartate and glutamate metabolism. The same GOBP pathways were enriched in NAc FN vs. FC as in MC vs. FC.

In the VTA in the chronic nicotine administration and withdrawal group, the top three PPI network clusters are shown for the MC vs. FC comparisons (Figure 7A). Enriched KEGG and GOBP pathways were ribosomes and translation, respectively, in cluster 1. In cluster 2, GABAergic synapse, morphine addiction, and glutamatergic synapse KEGG pathways and signal transduction GOBP pathways were enriched. In cluster 3, GOBP pathways of substantia nigra development, myelination, and response to toxic substance were enriched. Similarly, in the VTA chronic FN vs. FC comparison, cluster 1 had ribosome KEGG pathway enrichment as well as translation and RNA processing GOBP pathways (Figure 7B). In cluster 2, PPAR signaling KEGG pathway and protein oxidation and negative regulation of lipase activity GOBP pathways were enriched.

## Discussion

In this study, we analyzed whole tissue homogenates from VTA and NAc shell brain punches in male and female mice with and without nicotine administration. We not only examined differential protein expression between sexes under control conditions to analyze baseline sex differences, but we also investigated and compared the effect of nicotine within sex. An advantage of this experimental design is the use of two nicotine administration schedules, two mouse strains, and two sexes for proteomic comparisons. The breadth of these experimental manipulations not only allows for comparisons between nicotine reward and withdrawal, but also between strains, which is a known but underreported source of variability (Damaj et al., 2003; Bilkei-Gorzo et al., 2008; Isiegas et al., 2009).

In comparison to the number of proteins regulated after nicotine exposure in each sex, the number of proteins differentially expressed between sexes under control conditions were similar in C3H/HeJ mice and much greater in C57BL/6J mice. Other proteomic studies examining sex as a variable have also shown equivalent if not greater amounts of differential protein expression based on sex compared to other experimental manipulations (Liang et al., 2018; Sowers et al., 2019; Valent et al., 2019). Between our study and that of Sowers et al. (2019), which was also conducted in C57BL/6J mice but in hippocampi, there were several proteins that were differentially regulated in the same direction by sex (Alb, Camk2a, Cpne6, and Synpr) and some proteins that were differentially regulated but in opposite directions (Anxa6, L1cam, Scg2, and Vat1l). Other studies examining sex differences in experimentally naïve rodents (i.e., under “baseline” conditions), focused on specific neuronal compartments such as the synapse (Distler et al., 2020), microglia (Guneykaya et al., 2018), mitochondria in microvasculature (Cikic et al., 2021), or a specific type of protein modification (Khaliulin et al., 2020). Thus, the current study demonstrating a significant degree of sex difference in the mesolimbic proteome at baseline is an important contribution to broadening the understanding of baseline sex differences in the mouse brain.

### Enriched Pathways

The KEGG pathways enriched in MC vs. FC comparisons in the VTA and NAc shell of C3H/HeJ mice, and in the VTA of C57BL/6J mice, were many of the same pathways observed for the effect of nicotine within sex. These KEGG pathways were related to dopaminergic signaling, GABAergic signaling, calcium signaling, and neurological disorders. Pathways not specific to neurons were also enriched, such as certain metabolic pathways and ribosomal terms. Both the STRING analyses of each pairwise comparison and the further step of PPI sub-network analyses in Cytoscape and DAVID databases supported these findings. Importantly, these results suggest that the pathways exhibiting sex differences at baseline are relevant for neuronal function and for sex differences in the effects of nicotine.

The experiments that comprise this study also provide complementary and converging evidence for the breadth of sex differences in nicotine’s effects. In the VTA and NAc shell after sub-chronic nicotine administration the total numbers of proteins that were significantly altered by nicotine in pairwise comparisons within sex were similar. However, only ∼20% of those proteins were identical and altered in the same direction, and the STRING analyses showed divergent patterns between sexes. The KEGG pathways enriched in the VTA proteins upregulated after sub-chronic nicotine in female mice were similar to those KEGG pathways enriched in the proteins that were conversely downregulated after sub-chronic nicotine in male mice. Similarly, the KEGG pathways enriched in proteins downregulated after sub-chronic nicotine in female mice were more similar to the KEGG pathways enriched in proteins upregulated after sub-chronic nicotine in male mice; these KEGG pathways were more closely related to dopaminergic signaling, including dopaminergic synapse, tyrosine metabolism, and amphetamine addiction. In the NAc after sub-chronic nicotine administration, these dopamine signaling-related KEGG pathway terms were enriched in the proteins that were either upregulated or downregulated in FN vs. FC. However, the proteins representing these enriched pathways were more specific to dopaminergic function in the upregulated proteins (Th, Maob, and Prkaca) than in the downregulated proteins (Camk2d, Camk2g, Gng4, and Stx1a). In males, dopaminergic signaling-related KEGG pathway terms were enriched for the NAc shell proteins that were downregulated after sub-chronic nicotine exposure. The results suggest that a rewarding, sub-chronic schedule of nicotine administration produces sex- and brain region-dependent differences in dopaminergic signaling. Unlike baseline sex differences, for which the results of STRING and Cytoscape network analyses converged, the Cytoscape analyses for the effects of nicotine within sex were less informative of dopaminergic changes than the STRING analyses. Thus, based on the STRING analyses, sub-chronic nicotine administration in female mice increased dopaminergic signaling proteins in the NAc shell but decreased them in the VTA, while producing the opposite pattern in male mice (i.e., decreased dopaminergic signaling in the NAc shell and increased in the VTA).

These results appear consistent with prior literature showing sex differences in the brain region-specific regulation of dopaminergic signaling. For example, striatal dopamine release is differentially affected by nicotine and estrogen in male and female gonadectomized mice, in which estrogen increased nicotine-induced dopamine release in female striatal tissue and decreased it in male striatal tissue (Dluzen and Anderson, 1997). Decreased dopamine signaling-related proteins in the VTA of female mice after sub-chronic nicotine exposure may reflect the effect of enhanced inhibitory autoreceptor signaling. Related, in human smokers, higher D2-type autoreceptor availability was found in the midbrain of female smokers vs. non-smokers, but not in male smokers vs. non-smokers (Brown et al., 2012; Okita et al., 2016). Of note, male and female mice in our sub-chronic experiment were trained with different nicotine doses. The different doses produce similar nicotine reward, such that the sex differences observed in our study likely reflect divergent mechanisms underlying a convergent behavioral output. However, we cannot rule out the possibility that the sex differences partly reflect the effect of differing nicotine doses.

In the chronic nicotine administration and withdrawal experiment, there were two notable contrasts to the sub-chronic nicotine administration experiment in the VTA. First, there were 10 times more proteins significantly altered by chronic nicotine treatment and withdrawal in female than in male VTA, whereas similar numbers of proteins were differentially regulated in each sex by sub-chronic nicotine. Second, unlike in the sub-chronic nicotine experiment, similar KEGG pathways related to dopaminergic function and addictions were enriched in the proteins upregulated after nicotine withdrawal in both female and male mice. However, one major KEGG pathway enriched in FN vs. FC but not MN vs. MC was the ribosome, represented by almost a third (15/48) of the significantly upregulated proteins in the female nicotine group. This upregulation could represent an adaptive mechanism. For example, following developmental stress exposure, females appear to exhibit greater adaptability based on the increased expression of proteins related to protein synthesis and energy metabolism (Valent et al., 2019). Alternatively, increased ribosomal proteins in females may indicate increased VTA neuronal activity in response to acute stress (Holly and Miczek, 2016; Brischoux et al., 2009; Picciotto and Kenny, 2013) or more specifically to nicotine withdrawal, such as in corticotropin releasing factor (CRF) projections from VTA to the interpeduncular nucleus (Grieder et al., 2014; Zhao-Shea et al., 2015). These data support the theory that stress and withdrawal from nicotine are more important for driving nicotine addiction in females (Torres and O’Dell, 2016; O’Dell and Torres, 2014). Further, the results indicate that the VTA, and likely not the NAc shell, is an important node in the circuitry underlying the effects of nicotine withdrawal.

### Individual Proteins

An analysis of the proteins commonly altered across nicotine administration groups, sex, and strains revealed key proteins that may represent highly conserved mechanisms in nicotine addiction. Most consistently, GFAP and Ppp1r1b (DARPP-32) were significantly altered by nicotine reward or withdrawal and between sex, especially in the VTA.

Glial fibrillary acidic protein is a marker of astrocytes, which contribute to the blood-brain barrier, regulate neurotransmission and prevent neurotransmitter diffusion into extra synaptic spaces by active uptake of glutamate, glycine, and GABA, and are essential to synapse formation. Emerging research suggests that astrocytes, or glia in general, have more active roles in brain function than initially assumed (Barres, 2008; Paixao and Klein, 2010; Vijayaraghavan, 2009). For example, astrocytes express DAT and DA receptors, and thus may specifically regulate dopaminergic signaling (Fouyssac and Belin, 2019). Many studies show increases in GFAP after exposure to drugs of abuse such as cocaine (Fattore et al., 2002), morphine [which increased GFAP in the VTA but not the substantia nigra (SN); Beitner-Johnson et al., 1993; Goins and Bajic, 2018], and alcohol (where female mice showed more astrogliosis than males; Alfonso-Loeches et al., 2013). In a previous proteomic study after nicotine CPP (22 days of conditioning), GFAP was significantly reduced in rat hippocampus (Zhu et al., 2017). Others have reported that nicotine had no effect on GFAP in the hippocampal dentate gyrus (2 weeks of 0.1, 0.5 or 1 mg/kg, i.p.; Shingo and Kito, 2005), or in the NAc core after nicotine self-administration (Namba et al., 2020). However, GFAP was significantly reduced in a Western blot analysis of NAc core tissue after extinction and cue-induced reinstatement of nicotine self-administration (Namba et al., 2020).

In the current study, levels of GFAP were significantly altered in almost all pairwise comparisons. At baseline, GFAP was increased in male C3H/HeJ VTA, increased in female C57BL/6J VTA, and in female C3H/HeJ NAc shell. After sub-chronic nicotine, GFAP was decreased in the VTA of both male and female C3H/HeJ mice, whereas after chronic nicotine exposure and withdrawal it was increased in the VTA of both C57BL/6J sexes. Additionally, GFAP was decreased in NAc shell of female mice after sub-chronic nicotine. The direction of change was not uniform, suggesting that GFAP may be a more prominent player in the VTA than in the NAc shell, and that its regulation depends on sex and duration of nicotine administration.

Dopamine and cAMP-regulated phosphoprotein of 32 kDa is a phosphoprotein highly expressed in dopaminoceptive regions that can function as an inhibitor of protein phosphatase-1 (PP-1) or of protein kinase A (PKA), depending on its phosphorylation state (Svenningsson et al., 2004). Multiple phosphorylation sites on DARPP-32 are regulated by various neurotransmitters and drugs of abuse, including nicotine (Hamada et al., 2005, 2004; Walaas et al., 2011). Thus DARPP-32 plays a critical role in integrating signals received by dopaminoceptive neurons, and its phosphorylation states and downstream effects have been studied extensively (Svenningsson et al., 2004; Walaas et al., 2011). Nicotine regulates multiple DARPP-32 phosphorylation sites in striatal slices as well as *in vivo* after nicotine abstinence (Hamada et al., 2004, 2005; Abdolahi et al., 2010). These studies did not show alterations in total DARPP-32 protein levels, although other drug treatments such as alcohol, methylphenidate, and cocaine can regulate total DARPP-32 protein levels (Lynch et al., 2007; Souza et al., 2009; Abrahao et al., 2014). In the current study DARPP-32 was significantly increased in the VTA of MC vs. FC mice in both mouse strains, and in the NAc of MC vs. FC C3H/HeJ mice. Both sub-chronic and chronic nicotine and withdrawal also increased DARPP-32 in the VTA of both male and female mice, possibly reflecting proteins in the terminals of striatal projection neurons to VTA. DARPP-32 was not altered in the NAc where it is more abundant, but it is possible that the protein change reflects increased transport to terminals in the VTA. While DARPP-32 abundance was uniformly increased in the VTA, the functional impact may vary between sex or nicotine administration based on the phosphorylation state of the protein.

The findings shown here also point to potentially novel mechanisms underlying nicotine addiction. For example, beta-alanine metabolism was downregulated in male NAc shell and upregulated in female NAc shell after sub-chronic nicotine administration. Beta-alanine is an endogenous ligand of glycine receptors and has been implicated in regulating dopamine release in the striatum in response to alcohol, nicotine, and Δ9-tetrahydrocannabinol (Ericson et al., 2010; Jonsson et al., 2014). Although glycine receptors have been reported to play a role in alcohol addiction (Soderpalm et al., 2017), less is known about their role in nicotine addiction or in sex differences related to addiction.

PDE2A and PDE10A, the two proteins comprising the morphine addiction KEGG pathway that was upregulated in male VTA after chronic nicotine and withdrawal, can modulate dopaminergic signaling but have not been linked to nicotine addiction directly (Lin et al., 2010; Mango et al., 2014). Both proteins have been localized to striatal neurons, both at their cell bodies and axon terminals, with PDE10A preferentially enriched in basal ganglia circuits (Stephenson et al., 2012; D’Angelo et al., 2017), Interestingly, PDE10A inhibition can accelerate extinction of morphine CPP (Mu et al., 2014) and attenuates reinstatement of alcohol self-administration in rats with a history of exposure to stress (Logrip et al., 2014). Both PDE2A and PDE10A inhibitors are being studied in the clinic as potential treatments for schizophrenia (Siuciak et al., 2006), Huntington’s disease (Giampa et al., 2010), Parkinson’s disease (Niccolini et al., 2015), and other movement and neuropsychiatric disorders (Nishi and Snyder, 2010; Menniti et al., 2006).

Another example of a finding in our analyses that could provide novel targets for future investigations is Arpp21, or regulator of calmodulin signaling (RCS), a cAMP-regulated phosphoprotein of 21 kDa (Ouimet et al., 1989; Rakhilin et al., 2004). RCS was upregulated in both male and female VTA after chronic nicotine administration and withdrawal. RCS, similar to DARPP-32, is mostly concentrated in dopaminoceptive regions (Ouimet et al., 1989; Becker et al., 2008). Also similar to DARPP-32, RCS’s phosphorylation state can be increased and decreased by D1 and D2 dopamine receptor activation, respectively (Tsou et al., 1993; Caporaso et al., 2000), and increased by cocaine and methamphetamine administration (Caporaso et al., 2000). RCS likely works in concert with, or in a manner dependent on, DARPP-32 in signal integration functions (Walaas et al., 2011; Nair et al., 2016).

### Limitations and Future Directions

An important consideration in examining changes in individual proteins is the sensitivity and specificity of the proteomic technique used. Isobaric labeling, such as with TMT as used in our study or iTRAQ, is a powerful tool for multiplexed sample analysis. One potential pitfall of isobaric labeling is its vulnerability to ratio compression due to co-fragmentation of precursor ions (Mertins et al., 2012). Although ratio compression can lead to less accurate reporter ion quantification, its uniformity means that detection of significantly regulated elements is not compromised (Mertins et al., 2012). Further, we took steps to attenuate ratio compression, including extensive sample fractionation and SPS-MS3 acquisition (Rauniyar and Yates, 2014).

There have been mixed findings on the possible inverse relationship between the number of channels in a multiplexed experiment and the number of peptides identified (Pichler et al., 2010; Pottiez et al., 2012). However, the increased multiplexing is not only beneficial for higher throughput of samples, but also improves signal to noise ratio by running more replicates simultaneously, thereby reducing variation from multiple runs, and by performing protein identification from precursor ions of the mixed sample (i.e., same peptides from different samples identified together) (Rauniyar and Yates, 2014). In this study, there were fewer proteins identified for NAc shell in C57BL/6J and after chronic nicotine, as well as in both VTA and NAc in C3H/HeJ mice and after sub-chronic nicotine. This effect is less likely to be a strain difference since there were many more proteins identified in the VTA of C3H/HeJ mice compared to the NAc of the same strain. There also was not a consistent relationship between the numbers of proteins identified and the two brain region across strains. The difference in numbers of proteins identified and quantified between datasets may reflect, in part, a difference in data quality. For example, a combination of mass spectra with a lower signal-to-noise ratio and conservative peptide or protein identification criteria may limit the number of proteins identified in database searches (Rejtar et al., 2004). Furthermore, differential peptide modifications or lengths may affect peptide identification (Pichler et al., 2010).

Another important consideration is the statistical and analytic techniques used to detect significant changes in protein abundance and to probe their functional relevance. In the current study, we used the s0 parameter in Perseus as a weighting factor to take into consideration both fold-change and *q*-value to determine significance cutoffs, increasing the likelihood of detecting biologically significant changes. One limitation of our statistical analysis may be the multiplicity of pairwise comparisons without corrections for multiple comparisons. However, such a correction may be too restrictive to perform in addition to the Benjamini-Hochberg corrections applied to each individual protein comparison. Further, our pathway analyses using the STRING database accounted only for protein identity and not quantitative protein levels. However, our findings were strengthened by additional PPI network analyses that provided converging evidence of enriched pathways.

Following the identification of differentially regulated proteins by nicotine and between sex using an unbiased proteomic method as presented here, more targeted methods may be used to investigate the regulatory (e.g., phosphorylation states) and behavioral significance of these findings. With respect to sex differences, in particular, the relationship between protein changes and behavioral output may be particularly important to investigate given the possibility for divergent mechanisms leading to convergent outcomes (Becker and Koob, 2016). The role of sex hormones would also be a key area of future investigations. For example, estrous phase in intact, cycling female rodents has been shown to affect dopaminergic neuron activity and cocaine CPP (Calipari et al., 2017), but not nicotine reward-related behaviors including self-administration (Donny et al., 2000) or CPP (Torres et al., 2009; Lee et al., 2020). Estrous phase has also been shown to affect GFAP levels in the rat interpeduncular nucleus (Hajos et al., 2000) and hippocampus (Arias et al., 2009), but this effect has not been investigated in the mesolimbic circuity or in the context of drug reward. Further, the significant findings of baseline sex differences lay a foundation for understanding sex-convergent and sex-divergent mechanisms of mesolimbic system function and related behaviors.

## Conclusion

Sex differences in the mesolimbic system proteome are significant under baseline conditions as well as in response to nicotine. The scale of baseline sex differences is at least equivalent, or in C57BL/6J mice much greater than, that of sex differences after nicotine exposure. After a rewarding sub-chronic administration of nicotine, dopaminergic signaling pathways were altered in opposite directions in male and female mice, such that they were increased in the NAc and decreased in the VTA of female mice, and decreased in the NAc and increased in the VTA of male mice. These findings support previous literature on sex differences in primary reinforcement vs. regulation of negative affect driving the development of nicotine addiction in males vs. females, respectively. Further, the disproportionate protein regulation identified in female compared to male VTA after chronic nicotine and withdrawal suggests a greater effect of withdrawal in females, which might also explain sex differences in response to stress and rates of relapse to smoking in human tobacco users. Finally, this study compared the proteome across two sexes, two nicotine administration paradigms, and two mouse strains. Despite the breadth of experimental conditions and the hundreds of unique proteins that were differentially regulated, two proteins were repeatedly identified as significantly altered in sex and nicotine group pairwise comparisons, suggesting that GFAP and DARPP-32 are key proteins regulating the response to nicotine in male and female mice, especially in the VTA. Other unique pathways and proteins as identified in the data suggest novel targets for further investigation.

## Data Availability Statement

The datasets presented in this study can be found in online repositories. The names of the repository/repositories and accession number(s) can be found below: https://www.ebi.ac.uk/pride/, PXD023859.

## Ethics Statement

The animal study was reviewed and approved by Yale University IACUC.

## Author Contributions

AL, MM, AN, and MP conceived and designed the study. AL performed all mouse work. MM prepared samples for proteomic analysis. RW and TL performed mass spectrometry experiments. MM and AL performed statistical analyses. AL wrote the first draft of the manuscript, with writing contributions from MP, MM, and AN. All authors contributed to the manuscript revision, read, and approved the submitted version.

## Conflict of Interest

The authors declare that the research was conducted in the absence of any commercial or financial relationships that could be construed as a potential conflict of interest.

## Footnotes

1 https://www.who.int/news-room/fact-sheets/detail/tobacco

2 https://string-db.org/
